# Inspiratory Off-Switch Mediated by Optogenetic Activation of Inhibitory Neurons in the preBötzinger Complex In Vivo

**DOI:** 10.3390/ijms22042019

**Published:** 2021-02-18

**Authors:** Swen Hülsmann, Liya Hagos, Volker Eulenburg, Johannes Hirrlinger

**Affiliations:** 1 Department for Anesthesiology, University Medical Center, Georg-August University, Humboldtallee 23, D-37073 Göttingen, Germany; liya.hagos@stud.uni-goettingen.de; 2 Department for Anesthesiology and Intensive Care, Faculty of Medicine, University of Leipzig, Liebigstraße 20, D-04103 Leipzig, Germany; Volker.Eulenburg@medizin.uni-leipzig.de; 3 Carl-Ludwig-Institute for Physiology, Faculty of Medicine, University of Leipzig, Liebigstr. 27, D-04103 Leipzig, Germany; johannes.hirrlinger@medizin.uni-leipzig.de; 4 Department of Neurogenetics, Max-Planck-Institute of Experimental Medicine, Hermann-Rein-Str. 3, D-37075 Göttingen, Germany

**Keywords:** neuronal control of breathing, glycine, GABA, respiratory network

## Abstract

The role of inhibitory neurons in the respiratory network is a matter of ongoing debate. Conflicting and contradicting results are manifold and the question whether inhibitory neurons are essential for the generation of the respiratory rhythm as such is controversial. Inhibitory neurons are required in pulmonary reflexes for adapting the activity of the central respiratory network to the status of the lung and it is hypothesized that glycinergic neurons mediate the inspiratory off-switch. Over the years, optogenetic tools have been developed that allow for cell-specific activation of subsets of neurons in vitro and in vivo. In this study, we aimed to identify the effect of activation of inhibitory neurons in vivo. Here, we used a conditional transgenic mouse line that expresses Channelrhodopsin 2 in inhibitory neurons. A 200 µm multimode optical fiber ferrule was implanted in adult mice using stereotaxic surgery, allowing us to stimulate inhibitory, respiratory neurons within the core excitatory network in the preBötzinger complex of the ventrolateral medulla. We show that, in anesthetized mice, activation of inhibitory neurons by blue light (470 nm) continuously or with stimulation frequencies above 10 Hz results in a significant reduction of the respiratory rate, in some cases leading to complete cessation of breathing. However, a lower stimulation frequency (4–5 Hz) could induce a significant increase in the respiratory rate. This phenomenon can be explained by the resetting of the respiratory cycle, since stimulation during inspiration shortened the associated breath and thereby increased the respiratory rate, while stimulation during the expiratory interval reduced the respiratory rate. Taken together, these results support the concept that activation of inhibitory neurons mediates phase-switching by inhibiting excitatory rhythmogenic neurons in the preBötzinger complex.

## 1. Introduction

Breathing is an unconscious, involuntary process controlled by neural networks in the central and peripheral nervous system. These neural networks continuously regulate breathing to ensure optimal tissue oxygen delivery and carbon dioxide elimination. Effective tissue O_2_/CO_2_ exchange is assured by a respiratory neuronal network located in the medulla and the pons [1,2]. Excitatory neurons in the preBötzinger complex (preBötC) are indispensable for rhythm generation and inspiratory activity [3,4,5,6], while neurons in the Bötzinger complex (BötC) and the pontine respiratory group, e.g., the Kölliker Fuse nucleus (KF), provide a negative control and are assumed to mediate the off-switching of inspiratory activity [1,7,8,9,10]. Nonetheless, blockade of synaptic inhibition has produced contradictory results, ranging from complete loss of rhythmic activity [11] to only subtle alteration of frequency and amplitude [12].

The role of inhibitory neurons in the control of breathing by the respiratory network also has been tested using optogenetic tools, again, with partially contradictory results, ranging from increase in activity to complete suppression of breathing [9,13,14,15,16,17]. In the present study, we addressed these discrepancies taking advantage of mice that express Channelrhodopsin 2 (Chr2) in inhibitory neurons targeted by GlyT2-promoter activity. Using different stimulation protocols and phase-dependent triggering of inhibitory neuronal activity, we demonstrate that inhibitory neurons of the preBötC complex are involved in the inspiratory off-switch and phase transition and thereby modulate the respiratory rate.

## 2. Results

### 2.1. Continuous Stimulation of Inhibitory Neurons in the preBötC in Anesthetized Mice

**Figure 1:**
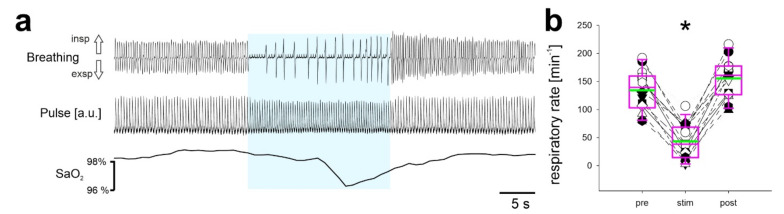
Continuous stimulation of inhibitory neurons in the preBötC reduces the respiratory rate. (**a**) The upper trace shows breathing movements recorded by the piezo-sensor (inspiration upward). The middle trace shows the pulse wave of the pulse oximetry and the lower trace shows oxygen saturation. (**b**) Quantification of respiratory rate (min^−1^) before (pre), during (stim), and after (post) the stimulation. *n* = 14 mice; * *p* < 0.05 (repeated measures (RM) ANOVA on Ranks).

In a first set of experiments, Chr2-expressing inhibitory neurons in the preBötC of anesthetized mice were continuously activated by constant application of light (Figure 1). This treatment induced a reduction of the respiratory rate from 134 ± 37 min^−1^ to 43 ± 32 min^−1^ (*n* = 14; *p* = 0.016). After cessation of light, the respiratory rhythm recovered, and the respiratory rate remained slightly increased (156 ± 35 min^−1^; *p* = 0.016; repeated measures (RM) ANOVA on Ranks). The reduction of the respiratory rate was accompanied by a significant increase of heart rate from 201 ± 57 min^−1^ to 226 ± 54 min^−1^ with stimulation (*p* < 0.001), which also recovered to 204 ± 57 min^−1^ after the end of the stimulation. This depression of breathing was accompanied by a mild reduction of the peripheral oxygen saturation (SpO_2_) from 93 ± 13% prior to stimulation to 88 ± 19% during stimulation (*n* = 14; *p* < 0.001; One Way RM ANOVA), which recovered quickly to 90 ± 16% (*p* = 0.178).

### 2.2. Stimulation Using Discontinuous Light Pulses with Different Frequencies

**Figure 2:**
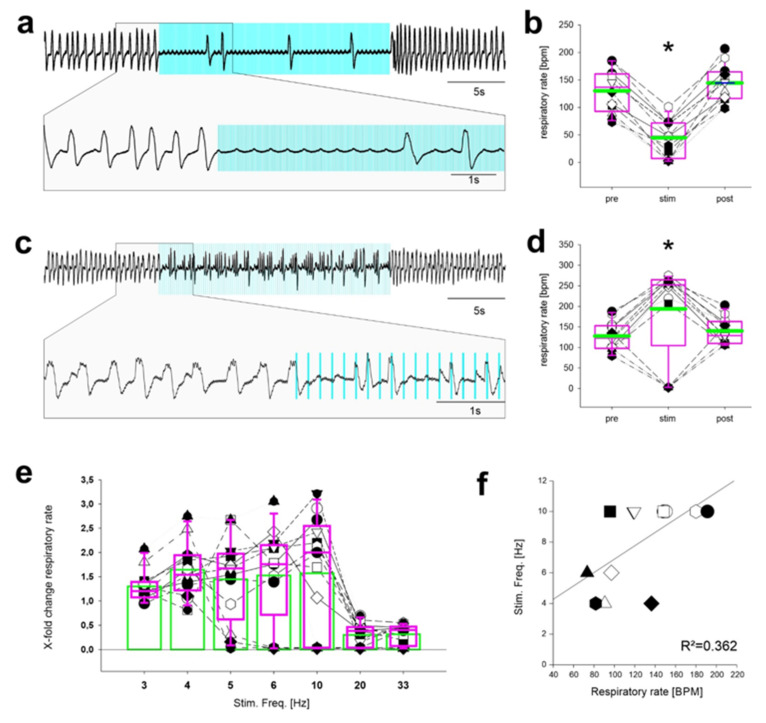
Effect of repetitive stimulation of inhibitory neurons in the preBötC depends on the stimulation frequency. (**a**) Thoracic chest wall movements recorded by a piezo-sensor (inspiration upward) and the pulse wave (gray trace). Repetitive 33 Hz stimulation (duration of each light pulse: 20 ms; blue bars). Asterisks show heart beat artifacts. (**b**) During 33 Hz light stimulation, the respiratory rate is reduced in all mice, yielding an overall statistically significant reduction during stimulation (* *p* < 0.001; *n* = 12; One way RM ANOVA). The rate recovered to a slightly higher frequency compared with the baseline level (*p* = 0.033). (**c**) Stimulation with a light pulse frequency of 6 Hz. The expanded trace shows that some deflections coincide with light pulses (‡; breaths), while others do not (*; heart beat). (**d**) Statistical analysis of 6 Hz stimulation reveals a significant increase in the rate of chest wall movement (*p* < 0.05: *n* = 13; RM ANOVA on Ranks). Of note, 3 mice showed a reduced respiratory rate. (**e**) Modulation of the respiratory rate by light stimulation with different stimulation frequencies. Data are from *n*= 7 mice and normalized to the pre-stimulus conditions (set as 1). (**f**) The stimulation frequency that leads to an increase in the respiratory rate depends on the baseline respiratory rate (*p* = 0.030, R^2^ = 0.362).

During physiological breathing, inhibitory neurons in the preBötC are not active continuously; therefore, continuous application of light does not closely recapitulate the physiological situation. To refine the model of activation of inhibitory neurons, trains of short light pulses (20 ms each pulse) were applied at different frequencies for a total duration of 20 s, uncovering a striking frequency dependence of the resulting breathing patterns (Figure 2). First, repetitive light pulses applied at the rather high frequencies of 33 Hz and 20 Hz resulted in respiratory suppression, which was similar to the effect observed during continuous application of light. Stimulation at 33 Hz reduced breathing from 130 ± 39 min^−1^ to 45 ± 33 min^−1^ (*n* = 12; *p* < 0.001; Figure 2a,b,e), with a recovery to 144 ± 34 min^−1^ (*p* = 0.033). Stimulation at 20 Hz had a similar effect (Figure 2e; 132 ± 37 min^−1^ prior to stimulation; 45 ± 40 min^−1^ during stimulation (*n* = 13; *p* = 0.012); 149 ± 31 min^−1^ after stimulation (*p* = 0.062; RM ANOVA on Ranks)). In contrast, at 6 Hz stimulation, some mice exhibited an increased respiratory rate while others showed a reduction of the frequency of respiratory thoracic movements (Figure 2c,d). Similar results were obtained for stimulation at 5 Hz or 10 Hz (Figure 2e). Furthermore, when the stimulation frequency was further reduced to 3 Hz and 4 Hz, the frequency of thoracic breathing movements was increased in all mice (Figure 2e). Of note, a significant although weak linear correlation (*p* = 0.030; R^2^ = 0.362) between the stimulation frequency—which leads to an increase in respiratory rate—and the baseline respiratory rate before the stimulation period was observed (Figure 2f).

### 2.3. Repetitive Stimulation of Inhibitory Neurons during Inspiration Increases the Respiratory Rate

**Figure 3:**
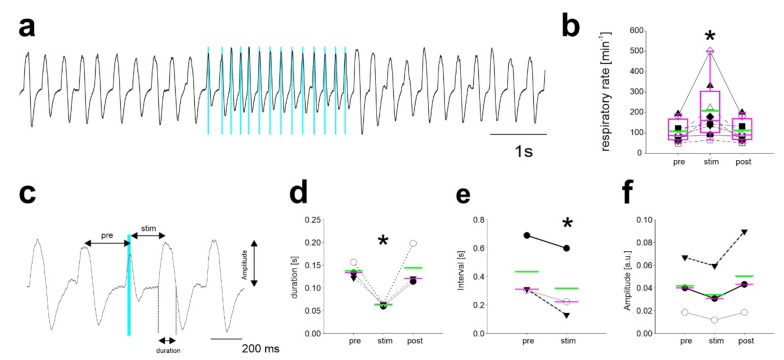
Stimulation during inspiration leads to an increase in the respiratory rate. (**a**) Thoracic breathing movements recorded with the piezo-sensor. Upward deflection indicates inspiration. Stimulation (blue bars, pulse length 20 ms) during inspiration leads to a decrease in the interval and, thus, (**b**) to an increase in the respiratory rate (* *p* < 0.001; *n* = 8; RM ANOVA on Ranks). After cessation of stimulation, the respiratory rate recovered to baseline levels. (**c**) Example trace showing the effect of a single light pulse during inspiration. (**d**) When the light pulse (blue bar in **c**) was applied during inspiration, the duration of the breath was reduced by the stimulation as compared with the pre-stimulus and the post-stimulus duration (* *p* < 0.05; One Way RM ANOVA; *n* = 3 mice). (**e**) The interval after the stimulation (stim) was also reduced as compared with the pre-stimulus interval (* *p* < 0.05; One-tailed paired *t*-test; *n* = 3 mice). (**f**) The breathing amplitude was, however, not significantly changed (*p* > 0.05; One way RM ANOVA; *n* = 3 mice). Lines in (**d**–**f**) show the median (magenta) and the mean (green).

The increased frequency of breathing despite experimental activation of inhibitory neurons could be due to entrainment and/or phase resetting of the respiratory rhythm by these inhibitory neurons. To test this hypothesis, activation of neurons by light pulses was performed at specific time points within the respiratory cycle. First, light pulses were applied repetitively in phase with inspiration (defined by the rising phase of the piezo-sensor signal; Figure 3a,b). During periods of inspiration-triggered light pulses, the respiratory rate was significantly increased from 109 ± 54 min^−1^ before to 209 ± 144 min^−1^ during stimulation (*n* = 8; *p* < 0.001; RM ANOVA on Ranks). After the end of the stimulation period, the respiratory rate returned to baseline levels (112 ± 54 min^−1^; *p* = 0.267).

To characterize the properties of this increase in respiratory frequency in more detail, light pulses were applied during inspiration in single respiratory cycles, thereby affecting only a single breath (Figure 3c). The duration of the ongoing respiratory cycle was reduced (63 ± 2 ms) as compared to the pre-stimulation cycle (137 ± 18 ms; *n* = 3; *p* = 0.035; Figure 3c,d). Moreover, the respiratory cycle after the stimulation was shorter (*n* = 3; one-tailed *t*-test; *p* = 0.0335; Figure 3e). The amplitude of the stimulated breath was not significantly different (0.04 ± 0.02 arbitrary units (au); *p* = 0.110; Figure 3e) compared to breaths before (0.04 ± 0.02 au) or after light application (0.05 ± 0.04 au).

**Figure 4:**
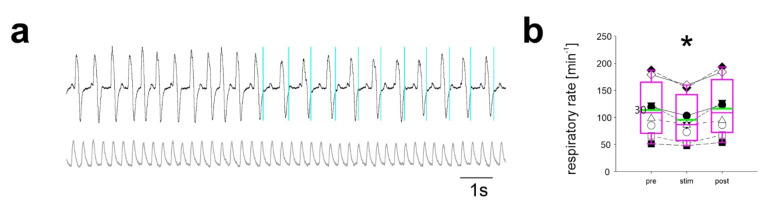
Stimulation during expiration leads to reduction in respiratory rate. (**a**) Breathing recorded with the piezo-sensor (gray trace: pulse signal measured by pulse oximetry). Stimulation with a 20 ms light pulse (blue bars) during expiration leads to an increase of the interval and, thus, (**b**) A reduction in the respiratory rate (* *p* < 0.001; One Way RM ANOVA; *n* = 8 mice).

In contrast, when the same light pulse was applied during expiration, the start of the following breath was delayed and the respiratory rate during the stimulation period was significantly reduced (Figure 4). The respiratory rate was 113 ± 49 min^−1^ before stimulation, 95 ± 42 min^−1^ during stimulation (*n* = 8; *p* < 0.001), and 116 ± 51 after the stimulation (*p* = 0.476).

In summary, these data provide evidence that the activation of inhibitory neurons of the preBötC during inspiration results in an off-switch of inspiratory neurons and thus a resetting of the cycle.

## 3. Discussion

The generation of the breathing rhythm, and particularly the contribution of specific types of neurons in the respiratory network, has been a subject of intense research in recent years [1,2,18]. State-of-the-art optogenetic approaches allow for modulating the activity of genetically defined populations of neurons with high temporal resolution, but have provided partially conflicting results when applied to the respiratory system [9,13,14,16,17]. Here, we demonstrate that modulation of the activity of the respiratory network induced by optogenetic activation of inhibitory neurons in the ventro-lateral medulla, which includes the central rhythm generator within the preBötC, crucially depends on the stimulation paradigm. Whereas continuous activation of these neurons led to suppression of breathing, repetitive stimulation with brief (20 ms) light pulses resulted in frequency-dependent deceleration or acceleration. Of note, the stimulation frequencies were in the range of the breathing frequency observed in the respective animals, suggesting different roles of the activity of glycinergic neurons depending on the phase of the respiratory cycle. Consistent with this hypothesis, brief light pulses applied during inspiration shorten the delay before the start of the next cycle, whereas activation during expiration caused a slowing of respiration by an onset-delay of the next cycle.

Continuous high frequency optogenetic stimulation of presumptive glycinergic preBötC neurons in the “working heart brainstem preparation”, reduces the respiratory rate; however, stimulation of these glycinergic neurons does not induce robust entrainment of the respiratory rhythm [13]. The GlyT2-Cre mice used in the present study target—in addition to glycinergic neurons—also at least a subset of GABAergic neurons [19,20], resulting in Chr2 expression in both glycinergic and GABAergic inhibitory neurons. Therefore, the additional optogenetic activation of GABAergic neurons might be critical for phase resetting. Indeed, termination of inspiration by activation of inhibitory neurons was also found in mice that utilized the promoter of the vesicular GABA transporter (Slc32A1, vGAT; also known as vesicular inhibitory amino acid transporter, VIAAT) to drive expression of ChR2 in inhibitory neurons [9]. However, this gene is expressed in all GABA- and glycinergic neurons, precluding an unequivocal conclusion on the type of inhibitory neuron responsible for phase resetting. Interestingly, an increase in the respiratory rate was observed with low-intensity optogenetic stimulation [9], a phenomenon that was not observed in our mice (data not shown). Such an increased respiratory rate with low-intensity stimulation is compatible with the activation of a GABAergic neuron population that mediates disinhibition of the excitatory kernel of the respiratory network via a population of glycinergic and/or GABAergic neurons with a higher stimulation threshold. Thus, higher intensities will result in a reduction in respiratory rate as shown in other models of Chr2-expression in inhibitory neurons [14,16,17]. Therefore, these divergent observations are most likely caused by differences between the targeted neuron populations in the different mouse lines as well as by different levels of ChR2-expression and activation thresholds.

When inhibitory neurons were stimulated by light application phase-locked to inspiration, a faster respiratory rate was observed (Figure 3), which is in line with the concept that inhibitory neurons mediate the off-switching of inspiration when activated during post-inspiration [21,22]. Mechanistically, it has been proposed that intrinsic membrane properties of preBötC neurons underlie the refractory period of the kernel and that activation of inhibitory neurons shortens this refractory period [14,23,24]. Moreover, because stimulation of the same population of neurons during expiration prolongs the current interval rather than inducing a new cycle, a simple rebound mechanism appears rather unlikely [15].

Inhibitory neurons are involved in many processes related to breathing, including local inhibition within the central rhythm generating circuit, but also mediating generalized sensory feedback like, e.g., the Hering–Breuer reflex. The optogenetic strategy used in this study does not allow for discriminating between neurons involved in these different tasks. It has been known since almost the beginning of respiratory research that vagotomy, and, thus, deafferentation of feedback from lung stretch receptors, decreases the respiratory rate [25]. Inhibitory neurons that mediate the vagal feedback are located in the dorsal parts of the medulla in the region of the nucleus of the solitary tract (NTS) and project to the preBötC [26,27]. We cannot exclude that during light stimulation in the ventral medulla (e.g., preBötC) axonal terminals of these neurons are also stimulated, and, thus, induction of action potentials in these inhibitory projection neurons might contribute to the observations of our experiments.

Optogenetic activation of inhibitory neurons suppresses the generation of respiratory rhythmic activity in the preBötC complex, as long as synaptic inhibition is sufficiently intense (high-intensity, continuous, or high-frequency stimulation) and regardless of which promoter is used to drive the expression of Chr2 [13,14,16]. Because inhibitory neurons project also to the contralateral preBötC, it is not surprising that unilateral preBötC stimulation also inhibits the respiratory rhythm [13], as illuminating the preBötC on one side will most likely also inhibit the contralateral preBötC by inducing action potentials in inhibitory neurons projecting to the contralateral side.

During normal breathing, different populations of inhibitory neurons are active in the three phases of the respiratory rhythm, but post-inspiratory (PI) neurons have in particular been discussed to mediate the inspiratory off-switch and phase shifting and suppression of inspiration [9,13,21,22]. Thus, activation of inhibitory neurons in the preBötC mimics the physiological effects of PI-activation. It is likely that optogenetic stimulation of a large number of these neurons can overcome the potentially disinhibitory effect of, e.g., early inspiratory preBötC neurons [1,9,28,29]. However, identifying genetic markers that allow for unequivocally differentiating between, e.g., early inspiratory neurons and post-inspiratory neurons will be a prerequisite to test this hypothesis experimentally.

We assume that stimulation of glycinergic neurons does not interfere with the Kölliker–Fuse nucleus (KF), which is also involved in phase-transition, since the KF-mediated inspiratory off-switch involves N-methyl-D-aspartate (NMDA) receptor-mediated glutamatergic transmission [30] rather than glycinergic neurons.

Technical Considerations: While the stimulation experiments were usually completed within less than 2 h after fiber implantation, we cannot formally exclude that the implantation procedure by itself might have influenced the experimental outcome, e.g., by some early alteration of the extracellular compartment along the path of the optical fiber caused by cell swelling, microglial invasion, or inflammation. However, since the optical fiber was not advanced directly into the preBötC (see methods), it can be assumed that damage by the fiber is vastly limited to areas of the brain that are not stimulated by light application. Furthermore, during optical stimulation small deflections in the piezo-sensor signal correlating with the pulse signal were observed. Therefore, these deflections most likely represent the heartbeat of the mouse. Of note, these deflections in the piezo-sensor signal preceded the peak of the pulse measured by pulse oximetry by approximately 50 ms. Assuming a distance between the heart and the thigh of adult mice of about 40 mm [31], an apparent pulse wave velocity of 0.8 m/s was calculated, which is very close to the data in the literature [32].

Conclusions: In summary, we here show that the outcome of optogenetically activating inhibitory neurons in the preBötC crucially depends on the experimental paradigm, specifically the time point of stimulation relative to the respiratory cycle. Our data support the concept that PI-neurons mediate phase-switching by inhibiting excitatory rhythmogenic neurons in the preBötzinger complex.

## 4. Methods

### 4.1. Ethics and Animal Handling

To generate transgenic mice that express Chr2 in inhibitory neurons in vivo, mice expressing Cre-recombinase under the control of the glycine transporter 2 promoter (Tg(Slc6a5-icre)121Veul [33]) were crossbred with 129S-Gt(ROSA)26Sor^tm32(CAG-COP4*H134R/EYFP)Hze/J^ mice that allow for Cre-dependent expression of Chr2 [34]. Breeding, handling, and all other experimental procedures were performed in compliance with the guidelines for the welfare of experimental animals issued by the European Communities Council Directive (2010/63/EU) and the German Protection of Animals Act (Tierschutzgesetz; TierSchG) and were approved by the Animal Welfare Commission of University Medical Center Göttingen, Germany and state authorities (LAVES).

**Figure 5:**
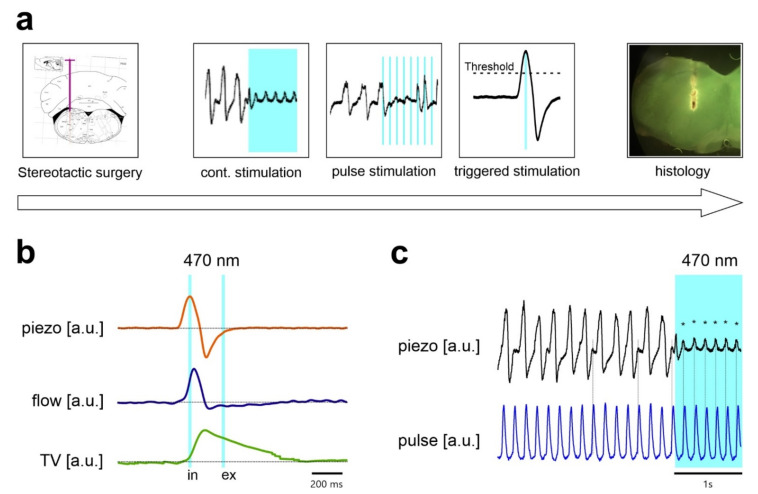
Experimental design and breathing measurement in anesthetized mice. (**a**) Time line of the experiments. After stereotactic implantation, the optogenetic stimulation was performed, starting with continuous stimulation, over repetitive stimulation (frequency between 33 Hz and 3 Hz) to threshold triggered stimulation. The experiment was concluded by transcardial perfusion for histology. (**b**) In an independent set of experiments, the signal of the piezo transducer was compared with plethysmography. The transducer was placed on the thorax of the mouse (piezo) with simultaneous measurement by whole-body plethysmography (the mouse was anesthetized by isoflurane). Flow trace (blue) and integrated flow representing tidal-volume (TV; green). Data are averaged from 20 cycles. Vertical blue lines indicate the approximate time point of the triggered light pulse. (**c**) Breathing movements (inspiration upward) detected by the piezo transducer. The piezo transducer also detects the beat of the heart (*), which becomes evident when breathing is suppressed by continuous activation of inhibitory neurons. Heart beats are synchronous with the signal output (blue trace) of the pulse oximeter.

Before surgical procedures and experimentation, mice received a subcutaneous (s.c.) injection of carprofen (5 mg/kg body weight (BW) dissolved in NaCl 0.9%). After 30 min, they were anesthetized by intraperitoneal (i.p.) injection of ketamine (200 mg/kg BW), Medetomidine (0.5 mg/kg BW), and Lidocaine (maximal 16 mg/kg BW). Depth of anesthesia was confirmed by the absence of the inter-digital nociceptive reflex response. Mice received supplementary O_2_ via a silicon tubing that was placed near the nose (0.2–0.4 L/min). Peripheral oxygen saturation (SpO_2_) was monitored with the MouseOx^®^ Plus Pulse Oximeter for rodents (Starr Life Sciences Corp., Oakmont, PA, USA). The analog pulse signal output (APSO) of the Pulse Oximeter was used to determine heart rate (Figure 5). For insertion of the optical fiber, mice were placed in a stereotactic frame (robot stereotaxic; Neurostar, Tübingen, Germany).

### 4.2. Monitoring of Breathing

Breathing was monitored with a piezo ceramic element (FT-31T-1.3A1-472; KEPO, Ningbo, China), which was placed underneath the thorax. In a separate experiment, the signal from the piezo-sensor was compared to changes in the lung volume during breathing (Figure 5). Therefore, mice anesthetized using isoflurane were placed on the piezo-sensor inside a whole-body plethysmography chamber that measures pressure changes resulting from the warming of the inspired air and cooling during expiration [35]. We used the chamber in the flow-through configuration [36] with a negative bias airflow of 150 mL†min^−1^. Pressure differences between the recording chamber and a reference chamber were captured by a DP103-12 pressure transducer (Validyne Engineering, Northridge, CA, USA) and passed through a sine wave carrier demodulator (CD-15, Validyne Engineering) for digitization (1 kHz sampling rate) with an analog–digital interface (PowerLab 8/35) and LabChart 8 software (ADInstruments, Spechbach, Germany). Prior to off-line analysis with LabChart software, the raw signal was band pass filtered off-line (0.5–20 Hz) to remove bias flow, artifacts, and noise, and then was integrated for the estimation of tidal volume. We used the standard integral settings of the “Integral Channel Calculation module” of the LabChart software, which utilizes all data points in a cycle and resets each time the source signal passes through zero to a positive value.

### 4.3. Stereotactic Implantation of Optical Fiber

A 200 µm diameter fiber optic cannula (CFMLC12L05; Thorlabs, Newton, NJ; 0.39 NA, length = 5 mm) was placed with the tip targeted to the preBötC using a custom-made holder that allowed us to have the patch cable (MAF2L1, Thorlabs) to be connected while approaching the area of interest. After setting the bregma and lambda for each individual mouse, head-tilt-correction was performed. The preBötC coordinates (Bregma −6.72 mm, 1.30 mm lateral [37]) were used by the Neurostar^®^ software for definition of the drill hole position (Ø 600 µm). We used the auto-stop drilling option of the software.

**Figure 6:**
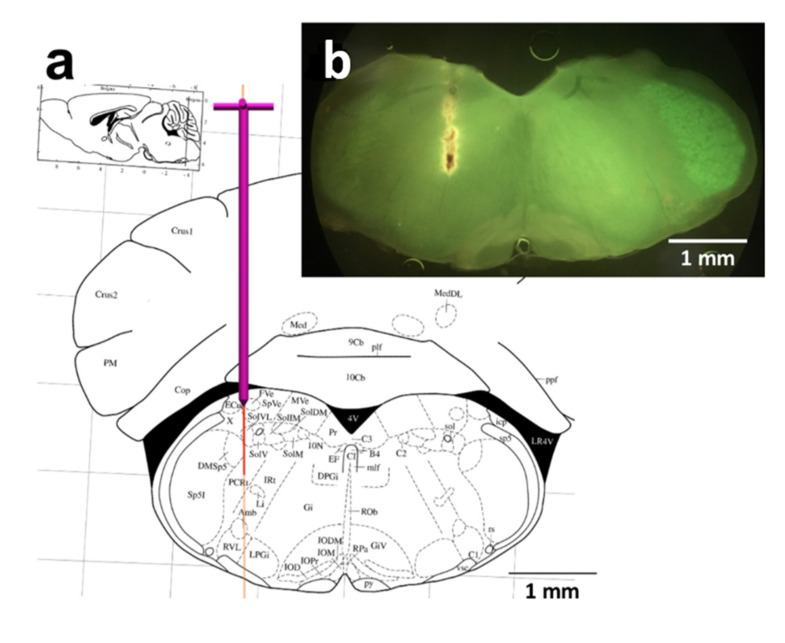
Positioning of the optical fiber (**a**) Screen copy of the NeuroStar^®^ software positioning window showing the planned tract of the fiber. (**b**) Image of the fiber position taken from a brain slice of the medulla after transcardial perfusion.

The tip of the optic fiber was placed dorsal to the preBötC (no further than the nucleus ambiguous) to illuminate the entire preBötC, allowing for activation of as many preBötC neurons as possible (Figure 6). With this setting, some rostral (BötC) or caudal (rVRG) neurons might also be activated. Fiber position was confirmed at the end of the experiment by histological examination of brain stem tissue slices (Figure 6). In some cases, the fiber was coated with fluorescent beads prior to the experiments for visualization of the fiber position in the fixed tissue.

Illumination at 470 nm was accomplished by a diode laser (LDU-01D, NPI electronics, Tamm, Germany) or by a light-emitting diode (LED) (FiberOptoMeter, NPI electronics), which was triggered by the digital output of an Axon™ Digidata 1440A Digitizer controlled by Axon™ pCLAMP™ software (Molecular Devices LLC., San Jose, CA, USA). Pulse protocols were generated using the by the pCLAMP™ software. Thoracic chest wall movement, heart rate, SpO_2_, and trigger signal were recorded with the Digidata 1440A Digitizer as well as with a PowerLab 8/35 (ADInstruments) and digitized using labchart 8. The fast response output add-on of LabChart/PowerLab was used to generate a digital output that is trigged by the breathing signal. The delay of the output signal was less than 1 ms.

### 4.4. Transcardial Perfusion for Tissue Fixation

For transcardial perfusion, mice were anesthetized by isoflurane. Deep anesthesia was confirmed by breathing pattern (gasping) and the absence of interdigital nociceptive reflexes. The chest was opened, the diaphragm cut, and the heart exposed. Mice were perfused transcardially with 50 mL of PBS followed by 100 mL of freshly prepared 4% paraformaldehyde in PBS (4% PFA), pH 7.4.

### 4.5. Microscopy and Image Acquisition

After transcardial perfusion, brains were post fixed in 4% PFA for at least 24 h. Medullary slices were cut at a thickness of 150 µm using a Leica VT1200s vibratome. After imbedding in Dako Fluorescence Mounting (Agilent), slices were placed under an upright epifluorescence microscope Examiner Z1 (Zeiss, Göttingen, Germany), illuminated with a SOLA SE lamp (Lumencor, Beaverton, OR, USA) and a dual band filter (EGFP/mCherry; AHF Analysentechnik AG, Tübingen, Germany). Images were captured with an iPhone SE (Apple, Cupertino, CA, USA), which was attached to the ocular with a smartphone adapter (Bresser GmbH, Rhede, Germany).

### 4.6. Data Analysis and Presentation

The PowerLab peak analysis algorithm was used to determine respiratory rate and other parameters from 20 s periods. Respiratory rate was calculated as breath per minute (min^−1^) from the averaged instant frequency (reciprocal of the peak-to-peak time interval). Statistical tests were performed using SigmaPlot (Version 14.0; Systat Software, San Jose, CA, USA). Significance of difference in respiratory measurements was accepted when *p* < 0.05. To determine whether significant effects of a particular stimulation protocol occurred, One-Way Repeated Measures (RM) Analysis of Variance (ANOVA) was used, followed by a multiple comparison versus control (Holm–Sidak method). In case the Equal variance test failed, a One-Way RM ANOVA on ranks was used with post hoc multiple comparisons versus control (Dunn’s method). A paired *t*-test was employed for comparison of intervals before and after stimulation. Correlation between parameter and effect (e.g., stimulation frequency for increase of respiratory rate and baseline respiratory rate) was tested using the “Linear Regression” tool of SigmaPlot. Pairwise Multiple Comparison Procedures were evaluated with a Tukey Test. In the summary figures, the boundary of the box closest to zero marks the 25th percentile, the magenta line within the box denotes the median, and the boundary of the box farthest from zero indicates the 75th percentile. Error bars above and below the box indicate the 90th and 10th percentiles, respectively. Green lines show the mean.

## Data Availability

The original contributions presented in the study are included in the article, further inquiries can be directed to the corresponding author.
